# Supplementary material for: Combined Metabolome and Transcriptome Analysis Highlights the Host’s Influence on Cistanche deserticola Metabolite Accumulation
Source: Int J Mol Sci. 2023 Apr 27;24(9):7968. doi: 10.3390/ijms24097968 (PMC10178529; doi:10.3390/ijms24097968)
Supplement: Supplementary file 1 [file ijms-24-07968-s001.zip › ijms-2286604-supplementary.pdf]

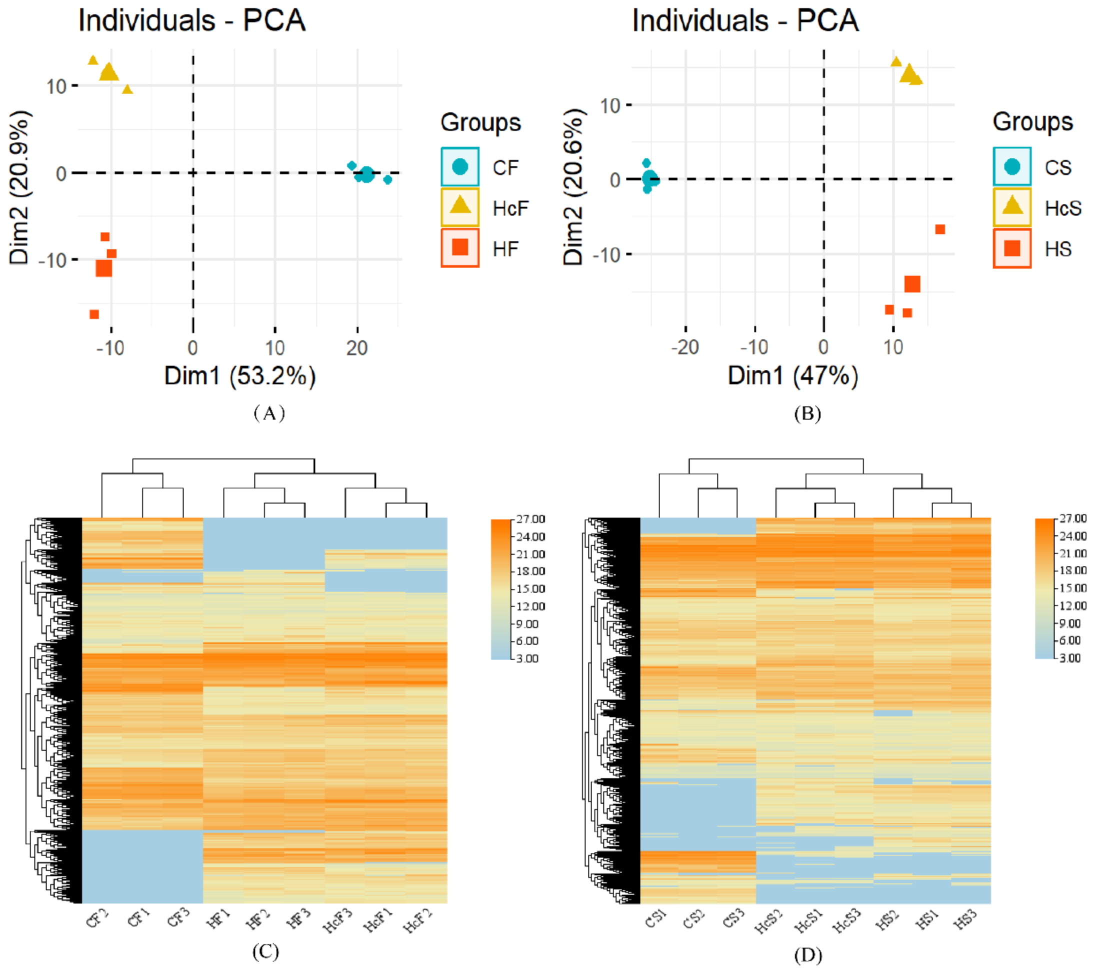

Supplement Figure S1. Clustering analysis based on metabolomic data. (A) Principal component analysis based on metabolomic data of CF, HcF and HF samples. (B) Principal component analysis based on metabolomic data of CS, HcS and HS samples. (C) Hierarchical cluster analysis based on metabolomic data of CF, HcF and HF samples. (D) Hierarchical cluster analysis based on metabolomic data of CS, HcS and HS samples.

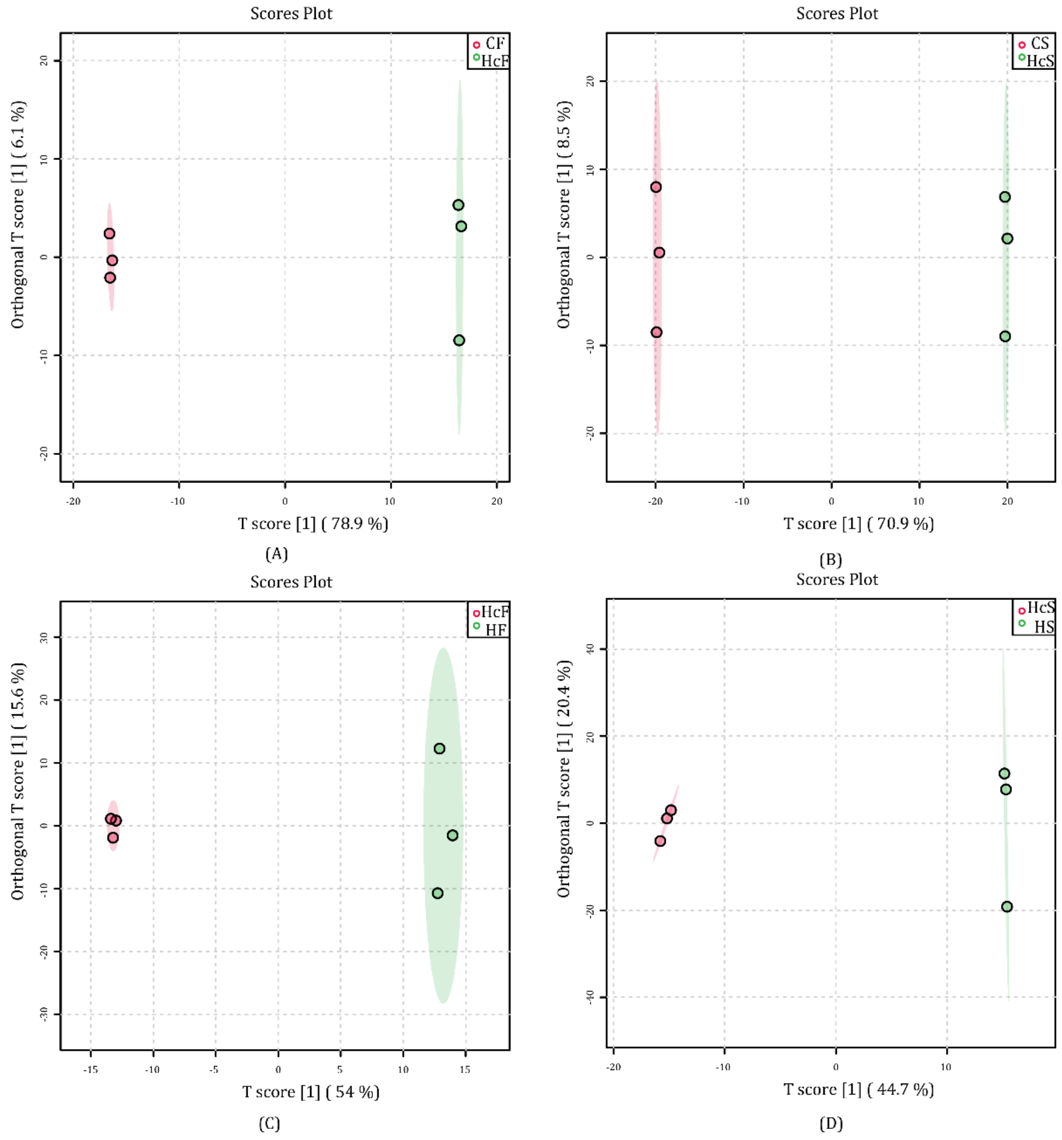

Supplement Figure S2. Orthogonal partial least squares discrimination analysis (OPLS-DA) based on metabolomic data. (A) OPLS-DA graph of CF-vs.-HcF group. (B) OPLS-DA graph of CS-vs.-HcS group. (C) OPLS-DA graph of HcF-vs.-HF group. (D) OPLS-DA graph of CS-vs.-HcS group.

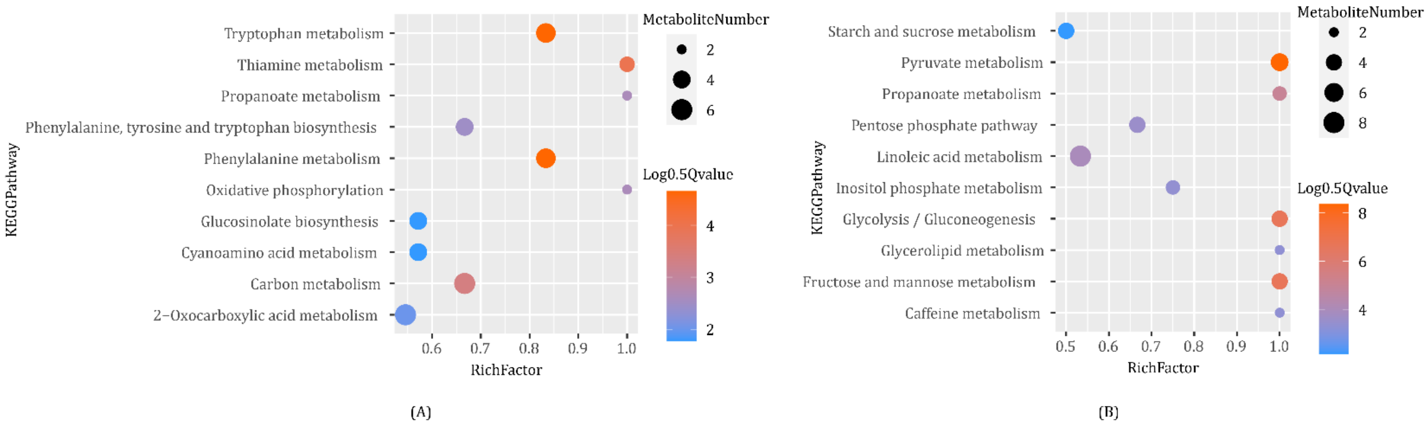

Supplement Figure S3. The bubble plots showed KEGG enrichment analysis results using *H. ammodendron* DAMs affected by *C. deserticola* parasitism. The bubble's color indicated q value and its size represented the number of DAMs enriched. (A) The bubble plot of top 10 KEGG pathways to which HcF-vs- HF DAMs were enriched. (B) The bubble plot of top 10 KEGG pathways to which HcS-vs- HS DAMs were enriched.

Supplement Table S1. Structures of PhGs in *C. deserticola* and *H. ammodenron*.

Compounds	R1	R2	R3	R4	R5	R6	R7
Isocrenatoside	OH	OH	O	O	Rha	Caf	H
Acteoside	OH	OH	H	H	Rha	Caf	H
Isomartynoside	OCH_3	OH	H	H	TA-Rha	H	Fer
Tubuloside B	OH	OH	H	Ac	TA-Rha	H	Caf
2'-Acetylacteoside	OH	OH	H	Ac	Rha	Caf	H
Salidroside	OH	H	H	H	H	H	H
Plantamajoside	OH	OH	H	H	Glc	Caf	H
Plantainoside A	OH	OH	H	H	Caf	H	H
Echinacoside	OH	OH	H	H	Rha	Caf	Glc
Cistanoside E	OH	OCH_3	H	H	Rha	H	H
Cistanoside C	OH	OCH_3	H	H	Rha	Caf	H
Cistanoside B	OH	OCH_3	H	H	Rha	Fer	Glc
Cistanoside A	OH	OCH_3	H	H	Rha	Caf	Glc
Brandioside	OH	OH	H	Ac	Rha	Caf	Rha
Osmanthuside H	OH	H	H	H	H	H	Api
Cistanoside H	OH	OH	H	Ac	Rha	H	H
Plantainoside C	OH	OH	H	H	Rha	H	Fer
Cistanoside D	OH	OCH_3	H	H	Rha	Fer	H
Epimeridinoside A	OH	OCH_3	H	H	Rha	H	Fer

Note: Ac, acetyl; Caf, trans-caffeoyl; Cm, trans-p-coumaroyl; Fer, trans-feruloyl; Glc, β-D-glucopyranose; Rha, α-L-rhamnopyranose; TA-Rha, 2′′, 3′′, 4′′-triacetyl-α-L-rhamnopyranose. Api, D-apiose. The general structure formula of phenylethanoid glycosides were presented below:

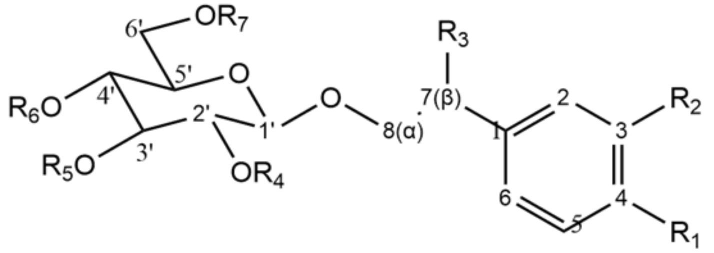

Supplement Table S2. Information of PhGs and their procusors in *C. deserticola* and *H. ammodendron*.

Mode	Compund	Formula	MW (Da)	CAS ID	RT (min)
Positive ion phase	Tyramine	C8H11NO	137.08	51-67-2	1.2
	Dopamine	C8H11NO2	153.08	51-61-6	1.11
	Phenylamine	C9H11NO2	165.07	63-91-2	1.99
	Tyrosine	C9H11NO3	181.07	60-18-4	1.13
	Isocrenatoside	C29H34O15	622.19	221895-09-6	3.98
	Cistanoside C	C30H38O15	638.22	94492-22-5	4.15
	Dopa	C9H11NO4	197.07	59-92-7	1.15
	Plantainoside A	C23H26O11	478.15	-	3.38
Negative ion phase	Tyrosol	C8H10O2	138.07	501-94-0	3.05
	Caffeic acid	C9H8O4	180.04	331-39-5	3.14
	Ferulic acid	C10H10O4	194.06	537-98-4	4.03
	Salidroside	C14H20O7	300.12	10338-51-9	2.63
	Cistanoside E	C21H32O12	476.19	97400-08-3	3.06

Acteoside	C29H36O15	624.21	61276-17-3	4.06
Plantamajoside	C29H36O16	640.2	104777-68-6	3.62
Cistanoside D	C31H40O15	652.24	94492-21-4	4.88
Isomartynoside	C31H40O15	652.24	94410-22-7	4.87
2'-Acetylacteoside	C31H38O16	666.22	94492-24-7	4.28
Tubuloside B	C31H38O16	666.22	112516-04-8	4.34
Echinacoside	C35H46O20	786.26	82854-37-3	3.31
Cistanoside A	C36H48O20	800.27	93236-42-1	3.58
Brandioside	C37H48O20	812.27	133393-81-4	3.84
Cistanoside B	C37H50O20	814.29	93236-41-0	3.86
Osmanthuside H	C19H28O11	432.16	149155-70-4	2.71
Cistanoside H	C22H32O13	504.18	104806-92-0	3.83
Plantainoside C	C30H38O15	638.22	136083-86-8	4.31
Epimeridinoside A	C31H40O15	652.24	848599-79-1	4.53
Cistanoside D	C31H40O15	652.24	94492-21-4	4.88

MW, molecular weight; RT, retention time.

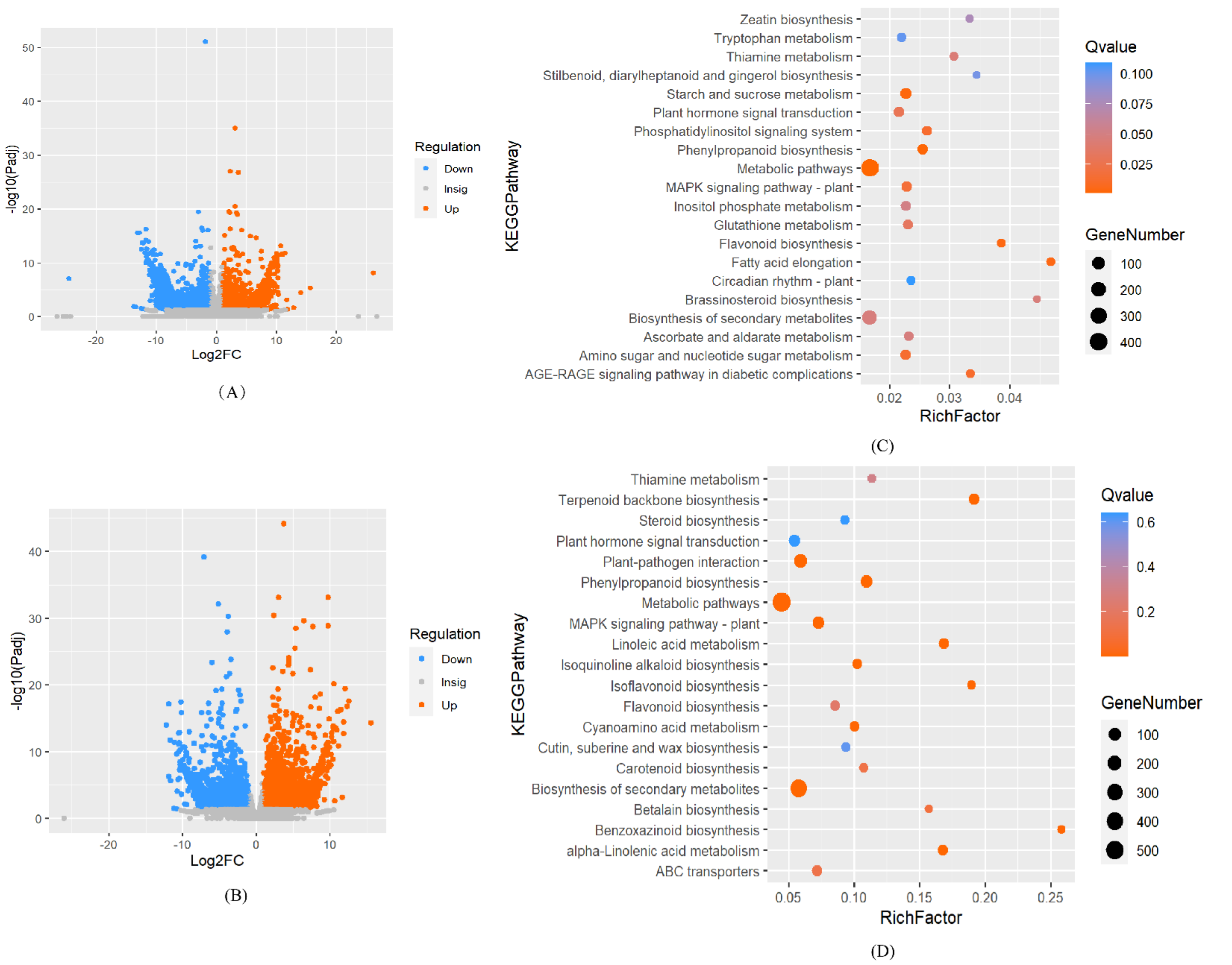

Supplement Figure S4. Transcriptomic changes in *H. ammodendron* roots caused by *C. deserticola* parasitism. The volcano plots visualized the filtering process of DEGs. Genes significantly downregulated (foldchange ≤ 0.5 and p value < 0.05) were marked by blue color while those significantly upregulated (foldchange ≥ 2 and p value < 0.05) were orange. The bubble plots showed KEGG enrichment analysis results using

DEGs. The bubble's color indicated q value and its size represented the number of DEGs enriched. (A) Volcano plot of genes expressing in HcF-vs.-HF group. (B) Volcano plot of genes expressing in HcS-vs.-HS group. (C) Bubble plot of top 20 KEGG pathways to which HcF-vs- HF DEGs were enriched. (D) Bubble plot of top 20 KEGG pathways to which HcS-vs- HS DEGs were enriched.

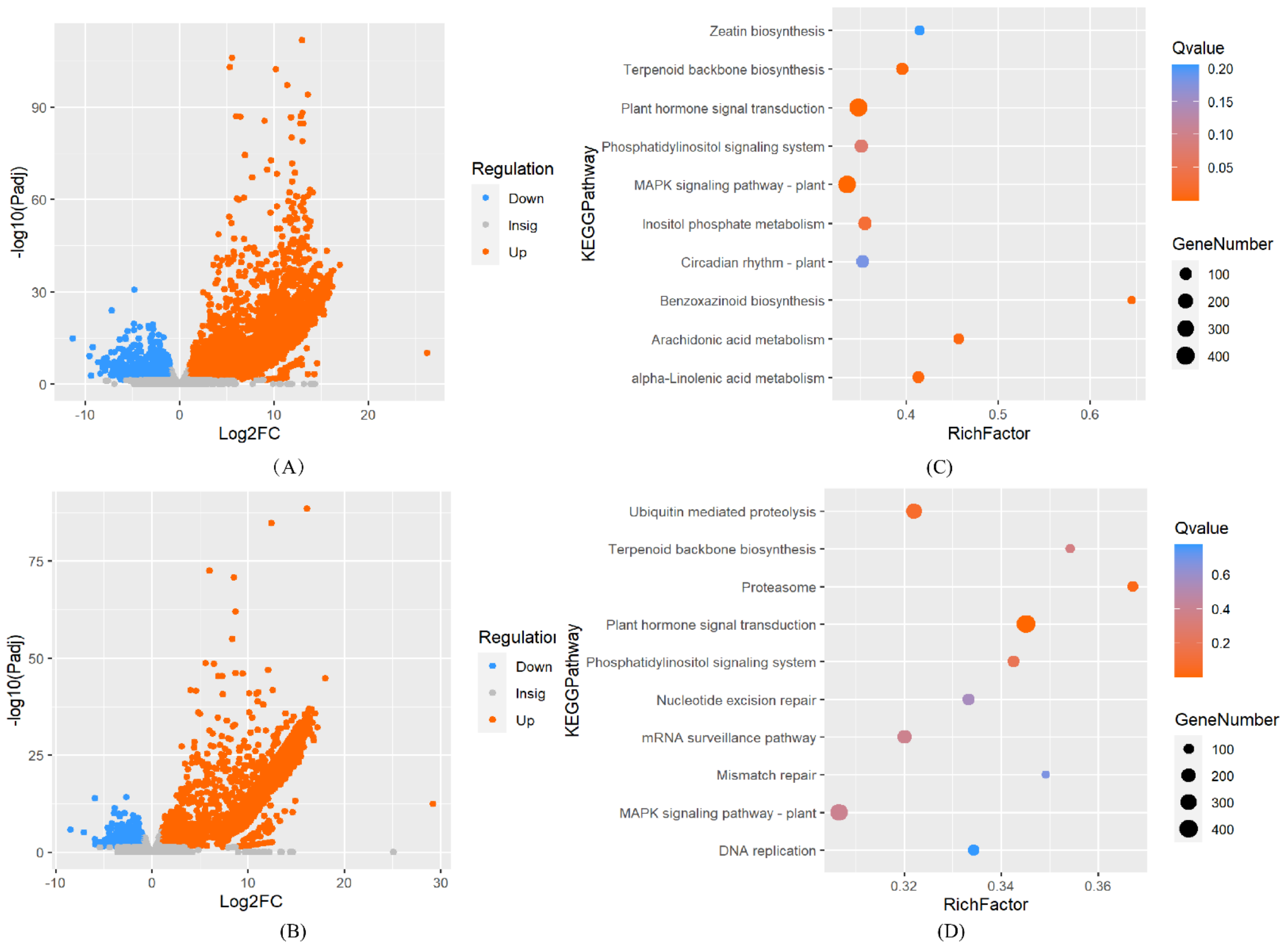

Supplement Figure S5. Transcriptomic changing pattern in the system composed by parasite, haustorium and host. The volcano plots visualized the filtering process of DEGs. Genes significantly downregulated (foldchange ≤ 0.5 and p value < 0.05) were marked by blue color while those significantly upregulated (foldchange ≥ 2 and p value < 0.05) were orange. The bubble plots showed KEGG enrichment analysis results using DEGs. The bubble's color indicated q value and its size represented the number of DEGs enriched. (A) Volcano plot of genes expressing in XS-vs.-CS group. (B) Volcano plot of genes expressing in XS-vs.-HcS group. (C) Bubble plot of top 10 KEGG pathways to which XS-vs.-CS DEGs were enriched. (D) Bubble plot of top 10 KEGG pathways to which HcS-vs.-HS DEGs were enriched.

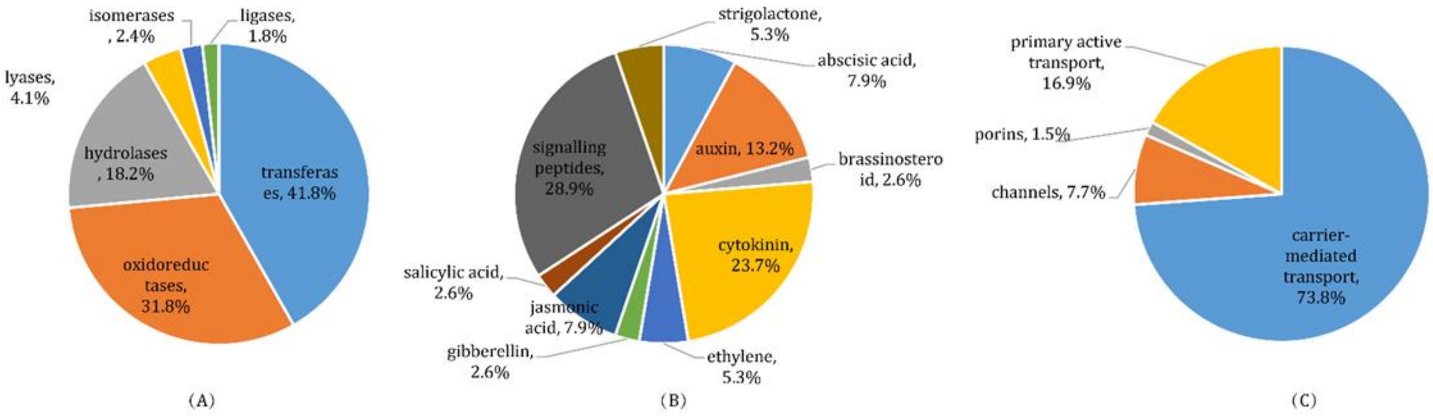

Supplement Figure S6. Mercator classifications of genes specifically upregulated in haustoria. Pie plots showed the percentages of genes annotated as 'enzyme classification' (A), 'phytohormone action' (B) and 'solute transport' (C).

Supplement Table S3 *H. ammodendron* PhG-synthetic DEGs affected by *C. deserticola* parasitism.

	GeneID	Log2(HcF-vs.-HF foldchange)	Log2(HcS-vs.-HS foldchange)
TyDC	Cluster-20042.57870	NA	-2.21298
	Cluster-20042.94036	NA	2.550736
PPO	Cluster-20042.64675	NA	-3.00593
	Cluster-20042.103369	NA	-5.03364
	Cluster-20042.60603	NA	-2.31877
	Cluster-20042.67782	NA	2.762793
	Cluster-20042.75123	NA	4.938769
PAO	Cluster-20042.99065	NA	2.107622
	Cluster-20042.49695	NA	2.919835
	Cluster-20042.69881	NA	3.163404
UGT	Cluster-65591.2858	8.146712	NA
	Cluster-57997.0	4.844912	NA
	Cluster-65591.172215	3.762266	NA
	Cluster-54039.0	-4.15679	NA
	Cluster-27477.1	1.859892	NA
	Cluster-65591.51912	1.353942	NA
	Cluster-65591.9408	-4.35132	NA
	Cluster-65591.165323	1.328536	NA
	Cluster-65591.67288	2.910998	NA
	Cluster-65591.30999	-1.40235	NA
	Cluster-43779.1	-10.1189	NA
	Cluster-20042.81537	NA	1.920222
	Cluster-20042.61118	NA	4.462931
	Cluster-20042.78674	NA	3.741149
	Cluster-20042.53531	NA	2.697838
	Cluster-20042.45312	NA	-3.89453
	Cluster-20042.100863	NA	2.590998
	Cluster-20042.103516	NA	-2.9074
	Cluster-20042.47888	NA	2.735516
	Cluster-20042.65342	NA	1.579276
	Cluster-20042.89184	NA	2.678052
	Cluster-20042.77086	NA	3.346782
	Cluster-20042.62811	NA	4.337779
	Cluster-20042.69314	NA	1.515049
	Cluster-20042.80697	NA	-1.27118
	Cluster-20042.109999	NA	-2.40483
	Cluster-20042.73576	NA	-1.06969
	Cluster-20042.64699	NA	2.18706
	Cluster-20042.32456	NA	-2.2045
	Cluster-20042.68255	NA	-1.56741
	Cluster-20042.75132	NA	1.112809
	Cluster-20042.58380	NA	-1.81437
	Cluster-20042.103245	NA	-4.73342

	Cluster-20042.54310	NA	2.054335
	Cluster-20042.65282	NA	1.127328
CCR	Cluster-65591.127998	-2.9585	NA
	Cluster-65591.62351	-1.22189	NA
	Cluster-65591.96138	-1.95595	NA
	Cluster-65591.99664	-1.11163	NA
	Cluster-65591.92661	-1.93125	NA
	Cluster-65591.62349	-1.2959	NA
	Cluster-65591.111775	-1.57694	NA
	Cluster-20042.63173	NA	1.12458
	Cluster-20042.91163	NA	-1.26916
	Cluster-20042.64779	NA	-1.27706
	Cluster-20042.55670	NA	-1.60013
	Cluster-20042.44943	NA	-1.62272
	Cluster-20042.114502	NA	-1.96643
	Cluster-20042.50091	NA	-2.3713
	Cluster-20042.104578	NA	-2.44877
	Cluster-20042.46762	NA	-2.89729
	Cluster-20042.117403	NA	-4.50519
	Cluster-20042.3913	NA	-4.63988
	Cluster-20042.144249	NA	-5.20393
PAL	Cluster-20042.78395	NA	3.693638
4CL	Cluster-65591.166393	2.160946	NA
	Cluster-20042.90539	NA	-1.76306
	Cluster-20042.99594	NA	-1.39321
	Cluster-31032.0	NA	8.764921
C4H	Cluster-65591.32894	2.511754	NA
	Cluster-65591.59422	1.326141	NA
HCT	Cluster-20042.83720	NA	-2.50542
	Cluster-5748.0	NA	4.832237
	Cluster-20042.106479	NA	-2.03028
C3H	Cluster-65591.81624	2.737275	NA
	Cluster-20042.44994	NA	3.823957

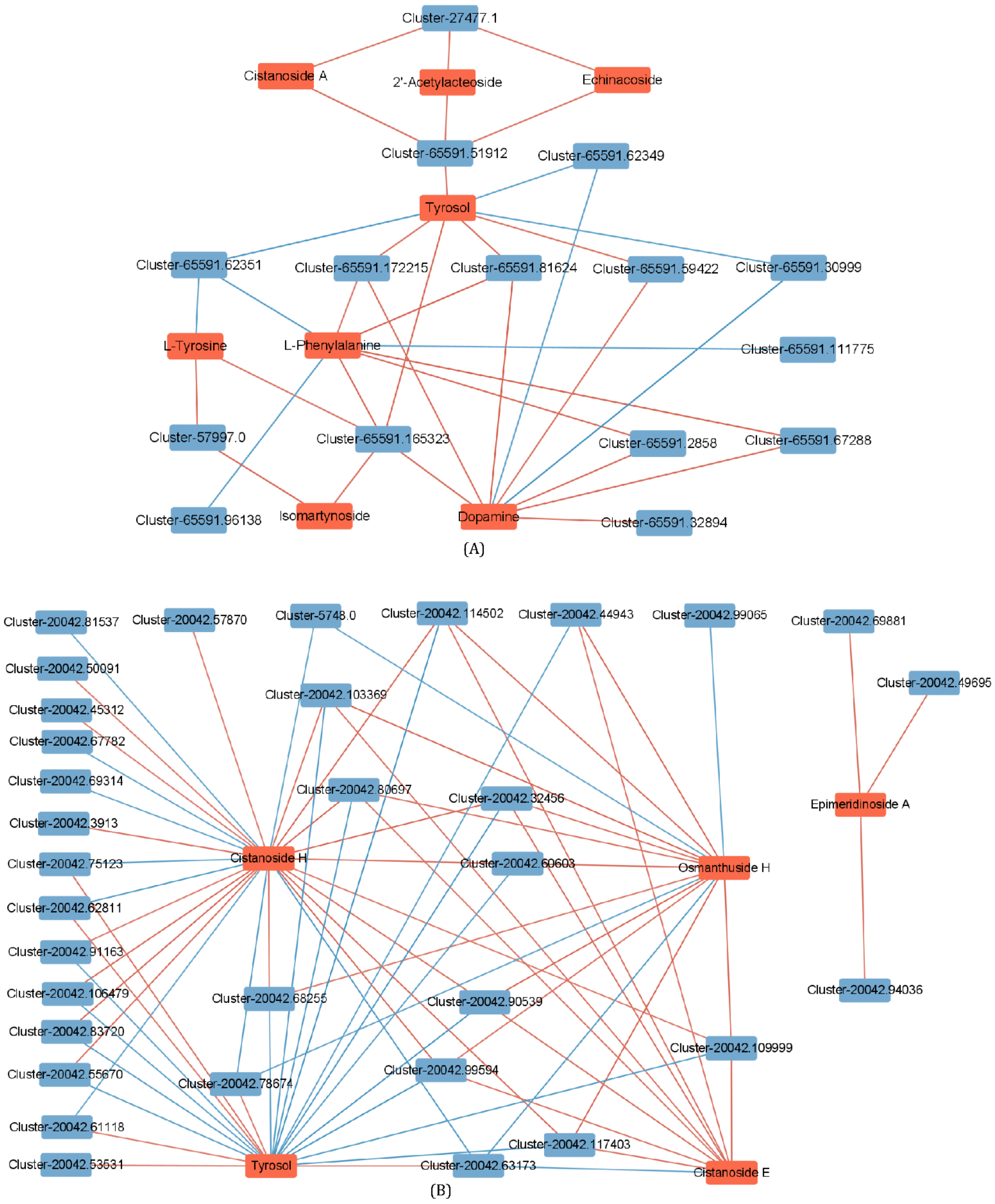

Supplement Figure S7. (A) Connection network between DEGs (blue grids) and DAMs (orange grids) in HcF-vs.-HF group. The blue lines indicate significant negative correlation (P<0.05, correlation coefficient< -0.8) and the orange lines indicate significant positive correlation (P<0.05, correlation coefficient > 0.8). (B) Connection network between DEGs (blue grids) and DAMs (orange grids) in HcS-vs.-HS group.

Supplement Table S4 UGT candidates in *H. ammodendron*

GeneID	Amino acid (aa)	Family	E-value	Identity (%)	Reference sequence ID
Cluster-21066.0	483	UGT92	1.45E-161	68.7	Q9LXV0
Cluster-32840.0	431	UGT92	3.75E-110	59.4	Q9LXV0
Cluster-54039.0	476	UGT92	1.51E-161	68.6	Q9LXV0
Cluster-65591.123403	356	UGT91	1.35E-125	68.1	Q9LTA3
Cluster-65591.123404	465	UGT91	3.99E-168	68.3	Q9LTA3
Cluster-65591.148827	443	UGT91	3.911E-83	51.9	Q9LTA3
Cluster-65591.159755	316	UGT91	3.194E-75	58.3	Q9LTA3
Cluster-65591.25693	391	UGT91	2.224E-90	56.5	Q9LTA3
Cluster-65591.75774	396	UGT91	6.073E-95	56.1	Q9LTA3
Cluster-65591.83451	316	UGT91	3.194E-75	58.3	Q9LTA3
Cluster-65591.83452	396	UGT91	6.073E-95	56.1	Q9LTA3
Cluster-65591.88153	396	UGT91	6.073E-95	56.1	Q9LTA3
Cluster-17228.0	451	UGT91	8.81E-136	63.5	Q940V3
Cluster-22345.0	453	UGT91	1.31E-133	63.8	Q940V3
Cluster-6556.0	453	UGT91	2.77E-134	64.2	Q940V3
Cluster-65591.105290	465	UGT91	2.02E-145	65.5	Q940V3
Cluster-65591.113604	465	UGT91	2.02E-145	65.5	Q940V3
Cluster-65591.53756	465	UGT91	2.02E-145	65.5	Q940V3
Cluster-65591.25486	455	UGT90	6.86E-157	68.4	Q9ZVX4
Cluster-65591.7404	455	UGT90	6.86E-157	68.4	Q9ZVX4
Cluster-44383.0	462	UGT89	6.08E-166	70.1	Q6VAA5
Cluster-44383.1	462	UGT89	6.08E-166	70.1	Q6VAA5
Cluster-65591.111737	386	UGT89	5.01E-130	68.1	Q9LZD8
Cluster-65591.16868	458	UGT89	2.63E-141	65.4	Q9LZD8
Cluster-65591.31200	473	UGT89	2.63E-154	67.2	Q9LZD8
Cluster-26255.1	394	UGT88	1.123E-47	48.0	Q6VAA7
Cluster-65591.80923	505	UGT88	5.78E-151	66.7	Q6VAA7
Cluster-65591.28176	308	UGT86	5.59E-101	68.3	Q9ZUV0
Cluster-29865.0	304	UGT86	7.05E-110	72.2	Q9SJL0
Cluster-47176.0	304	UGT86	2.14E-110	71.9	Q9SJL0
Cluster-52305.0	474	UGT86	4.67E-144	63.9	Q9SJL0
Cluster-65591.124228	488	UGT86	2.59E-179	70.1	Q9SJL0
Cluster-65591.134475	491	UGT86	1.93E-179	70.3	Q9SJL0
Cluster-65591.90511	484	UGT86	1.78E-178	70.7	Q9SJL0
Cluster-65591.90512	339	UGT86	4.26E-131	72.6	Q9SJL0
Cluster-65591.9406	484	UGT86	7.34E-179	70.9	Q9SJL0
Cluster-65591.9407	485	UGT86	6.83E-179	71.0	Q9SJL0
Cluster-65591.9408	487	UGT86	5.65E-179	71.2	Q9SJL0
Cluster-65591.79144	321	UGT85	4E-120	70.5	Q6VAB3
Cluster-65591.95218	321	UGT85	4E-120	70.5	Q6VAB3
Cluster-65591.160000	489	UGT85	0	71.8	Q9ZWJ3
Cluster-65591.14139	451	UGT83	1.77E-100	54.9	Q9SGA8
Cluster-65591.105881	311	UGT79	1.52E-104	69.4	Q9T080
Cluster-65591.105884	344	UGT79	2.4E-108	64.5	Q9T080
Cluster-65591.111516	431	UGT79	4.45E-141	64.7	Q9T080

Cluster-65591.137048	306	UGT79	5.85E-105	69.8	Q9T080
Cluster-65591.162455	340	UGT79	9.3E-109	65.0	Q9T080
Cluster-65591.105885	420	UGT79	1.8E-133	62.5	Q9XIQ4
Cluster-65591.105886	420	UGT79	1.8E-133	62.5	Q9XIQ4
Cluster-65591.105889	420	UGT79	1.8E-133	62.5	Q9XIQ4
Cluster-65591.105895	420	UGT79	1.8E-133	62.5	Q9XIQ4
Cluster-65591.15682	320	UGT76	8.626E-96	64.2	Q9C768
Cluster-65591.15686	314	UGT76	4.1E-88	62.7	Q9C768
Cluster-65591.162327	455	UGT74	5.52E-173	69.5	O22822
Cluster-65591.68313	359	UGT74	1.8E-128	69.3	O22822
Cluster-65591.69728	309	UGT74	1.262E-94	63.4	Q9SYK9
Cluster-65591.82796	464	UGT74	1.678E-52	48.9	Q9SKC5
Cluster-65591.67648	450	UGT74	6.25E-138	64.9	O48676
Cluster-65591.67649	450	UGT74	6.25E-138	64.9	O48676
Cluster-65591.28465	496	UGT73	0	76.0	Q9SCP6
Cluster-65591.74129	380	UGT73	3.635E-38	45.9	A0A2R4LMF9
Cluster-65591.83204	370	UGT73	4.601E-38	47.1	A0A2R4LMF9
Cluster-27477.1	474	UGT73	6.2E-142	62.8	Q9ZQG4
Cluster-45552.0	494	UGT73	2.23E-112	57.3	Q9ZQG4
Cluster-65591.115428	302	UGT73	5.805E-52	57.6	Q9ZQG4
Cluster-45498.0	489	UGT73	1.13E-115	58.1	Q7Y232
Cluster-65591.84717	352	UGT73	5.023E-54	60.7	Q7Y232
Cluster-27477.0	472	UGT73	1.52E-134	62.9	Q8W491
Cluster-37808.0	364	UGT73	3.24E-111	63.6	Q8W491
Cluster-65591.134604	415	UGT73	1.59E-115	62.2	Q8W491
Cluster-65591.142099	371	UGT73	1.31E-142	71.5	Q8W491
Cluster-65591.32140	324	UGT73	3.17E-123	71.6	Q8W491
Cluster-65591.84640	408	UGT73	1.89E-166	73.9	Q8W491
Cluster-65591.81750	386	UGT72	1.25E-145	70.3	Q9LNI1
Cluster-65591.90680	386	UGT72	1.25E-145	70.3	Q9LNI1
Cluster-65591.104312	467	UGT72	4.04E-171	68.0	Q9M156
Cluster-65591.123589	366	UGT72	3.36E-123	68.0	Q9M156
Cluster-65591.123590	367	UGT72	2.21E-123	68.0	Q9M156
Cluster-65591.123591	361	UGT72	9.11E-145	73.3	Q9M156
Cluster-65591.132635	366	UGT72	8.73E-124	68.3	Q9M156
Cluster-65591.67590	368	UGT72	1.63E-123	67.8	Q9M156
Cluster-65591.87505	472	UGT72	0	73.8	Q9M156
Cluster-65591.92058	472	UGT72	0	73.8	Q9M156
Cluster-65591.97292	367	UGT72	6.75E-123	67.6	Q9M156
Cluster-65591.23010	320	UGT71	1.634E-70	57.9	Q6VAB2
Cluster-65591.24510	328	UGT71	3.225E-85	61.4	Q6VAB2
Cluster-65591.65784	477	UGT71	2.33E-151	65.1	Q6VAB2
Cluster-65591.84842	324	UGT71	6.987E-72	58.5	Q6VAB2
Cluster-65591.93286	484	UGT71	1.87E-142	65.6	Q6VAB2
Cluster-65591.153685	495	UGT71	9.34E-153	64.9	D3UAG1
Cluster-65591.132161	487	UGT71	2.39E-151	64.7	D3THI6
Cluster-65591.137447	415	UGT71	3.73E-138	67.7	D3THI6
Cluster-65591.34619	450	UGT71	1.32E-117	60.3	D3THI6

Cluster-65591.126569	463	UGT88	9.964E-56	50.0	A0A067YB04
Cluster-43779.1	570	UGT80	2.22E-134	60.8	Q9XIG1
Cluster-65591.103241	497	UGT80	0	87.8	Q9M8Z7
Cluster-65591.103242	324	UGT80	0	89.3	Q9M8Z7
Cluster-65591.74649	588	UGT80	0	82.8	Q9M8Z7
Cluster-65591.92668	556	UGT80	0	87.7	Q9M8Z7
Cluster-65591.92669	497	UGT80	0	88.0	Q9M8Z7
Cluster-65591.53969	497	UGT75	4.49E-145	64.6	A7MAS5
Cluster-65591.67288	497	UGT75	3.28E-146	64.8	A7MAS5
Cluster-54362.0	449	UGT74	1.18E-149	67.4	K7NBW3
Cluster-65591.142893	467	UGT74	3.46E-119	60.3	K7NBW3
Cluster-65591.142894	467	UGT74	3.46E-119	60.3	K7NBW3
Cluster-65591.66190	458	UGT74	2.71E-152	67.6	K7NBW3
Cluster-65591.71660	449	UGT74	6.06E-149	67.4	K7NBW3
Cluster-65591.79512	458	UGT74	1.18E-152	67.6	K7NBW3
Cluster-65591.86205	458	UGT74	1.22E-152	67.6	K7NBW3
Cluster-62357.0	311	UGT85	1.9E-116	70.6	G3FIN8
Cluster-65591.87593	502	UGT84	0	78.8	A0A193AUF6
Cluster-57997.0	477	UGT85	0	75.5	F8WKW1
Cluster-65591.149863	382	UGT85	3.58E-157	74.7	F8WKW1
Cluster-65591.149864	407	UGT85	5.46E-176	76.0	F8WKW1
Cluster-65591.167954	470	UGT85	0	70.8	F8WKW1
Cluster-65591.19046	479	UGT85	0	70.0	F8WKW1
Cluster-65591.2858	477	UGT85	0	75.5	F8WKW1
Cluster-65591.80733	312	UGT85	4.37E-143	77.4	F8WKW1
Cluster-65591.81499	479	UGT85	0	70.4	F8WKW1
Cluster-65591.92026	312	UGT85	4.37E-143	77.4	F8WKW1
Cluster-65591.94515	490	UGT85	0	71.6	F8WKW1
Cluster-65591.170819	315	UGT709	4.24E-123	73.3	U3U992
Cluster-65591.170820	456	UGT709	2.88E-152	67.9	U3U992
Cluster-65591.171180	469	UGT709	5.5E-150	66.2	U3U992
Cluster-24476.0	431	UGT85	0	74.7	B2XBQ5
Cluster-9773.0	450	UGT78	8.43E-140	64.2	Q9LFJ8
Cluster-69206.3	312	UGT80	1.88E-154	81.6	Q9XIG1
Cluster-69206.7	335	UGT80	4.35E-177	83.7	Q9XIG1
Cluster-69206.8	398	UGT80	0	81.6	Q9XIG1
Cluster-10683.0	510	UGT80	1.19E-136	64.8	Q9M8Z7
Cluster-10684.0	494	UGT80	1.75E-136	64.8	Q9M8Z7
Cluster-24999.0	540	UGT80	2.32E-134	64.1	Q9M8Z7
Cluster-24999.1	585	UGT80	2.22E-134	64.1	Q9M8Z7
Cluster-24999.2	585	UGT80	2.22E-134	64.1	Q9M8Z7

Supplement Table S5. UGT candidates in *C. deserticola*.

GeneID	Amino acid (aa)	Family	E-value	Identity (%)	Reference sequence ID
Cluster-10691.114607	364	UGT90	7.1E-116	65.2	Q9ZVX4
Cluster-10691.117489	363	UGT90	4.779E-112	64.1	Q9ZVX4
Cluster-10691.146973	364	UGT90	8.265E-116	65.2	Q9ZVX4
Cluster-10691.210013	397	UGT90	2.162E-117	64.6	Q9ZVX4

Cluster-10691.39306	408	UGT90	9.395E-124	64.9	Q9ZVX4
Cluster-10691.39309	467	UGT90	1.933E-136	64.7	Q9ZVX4
Cluster-10691.110679	472	UGT89	9.017E-179	73.2	Q6VAA5
Cluster-10691.118503	475	UGT89	1.796E-180	73.2	Q6VAA5
Cluster-10691.121191	472	UGT89	9.017E-179	73.2	Q6VAA5
Cluster-10691.46129	439	UGT89	5.253E-169	71.3	Q9LZD8
Cluster-5739.0	470	UGT89	1.899E-162	68.7	Q9LZD8
Cluster-10691.139803	474	UGT88	1.011E-162	68.1	D3UAG7
Cluster-10691.131097	472	UGT88	9.781E-163	69.0	Q6VAA7
Cluster-10691.131098	472	UGT88	9.781E-163	69.0	Q6VAA7
Cluster-10691.185902	458	UGT88	4.814E-161	67.9	Q6VAA7
Cluster-10691.112663	458	UGT87	1.599E-160	70.0	O64732
Cluster-10691.121503	456	UGT87	1.567E-159	69.9	O64732
Cluster-10691.196067	314	UGT87	9.7074E-82	62.6	O64732
Cluster-10691.201967	314	UGT87	9.7074E-82	62.6	O64732
Cluster-10691.119149	453	UGT86	1.279E-164	69.7	Q9SJL0
Cluster-10691.151473	315	UGT86	4.223E-108	69.9	Q9SJL0
Cluster-10691.33814	354	UGT86	4.381E-122	69.3	Q9SJL0
Cluster-10691.74493	378	UGT86	7.369E-117	65.2	Q9SJL0
Cluster-10691.91766	315	UGT86	4.223E-108	69.9	Q9SJL0
Cluster-26714.0	407	UGT86	2.982E-111	60.6	Q9SJL0
Cluster-10691.146136	323	UGT85	1.162E-162	80.6	Q6VAB3
Cluster-10691.146137	347	UGT85	9.983E-179	80.8	Q6VAB3
Cluster-10691.162683	485	UGT85	0	73.0	Q9SK82
Cluster-10691.142628	307	UGT84	5.4862E-16	44.9	Q5XF20
Cluster-10691.164268	451	UGT79	5.433E-147	63.6	I1KEV6
Cluster-10691.164269	414	UGT79	5.257E-135	63.8	I1KEV6
Cluster-10691.205645	449	UGT79	1.594E-145	63.5	I1KEV6
Cluster-10691.225522	451	UGT79	2.237E-146	63.6	I1KEV6
Cluster-10691.225523	416	UGT79	1.205E-135	63.7	I1KEV6
Cluster-10691.123433	456	UGT79	2.277E-180	71.4	Q9T081
Cluster-10691.147537	457	UGT76	8.342E-165	69.0	Q9M052
Cluster-10691.188735	448	UGT76	8.387E-163	69.4	Q9M052
Cluster-10691.34936	456	UGT76	9.556E-164	69.5	Q9M052
Cluster-10691.121101	428	UGT74	3.734E-132	64.1	Q6VAA6
Cluster-13596.0	428	UGT74	3.734E-132	64.1	Q6VAA6
Cluster-10691.93238	467	UGT74	0	72.5	O22822
Cluster-10691.217659	307	UGT74	8.3607E-16	41.6	Q9SKC5
Cluster-10691.206681	338	UGT73	8.634E-145	75.6	Q9SCP6
Cluster-13956.0	370	UGT73	8.2756E-42	57.6	A0A2R4LMF9
Cluster-10691.99921	483	UGT73	6.414E-175	71.5	K4GGT4
Cluster-10691.130076	479	UGT73	1.085E-133	62.1	Q9ZQG4
Cluster-10691.166032	415	UGT73	1.3599E-51	60.4	Q7Y232
Cluster-13955.0	444	UGT73	1.0506E-54	59.7	Q7Y232
Cluster-10691.116093	479	UGT73	8.349E-133	61.6	Q8W491
Cluster-10691.93081	469	UGT72	0	76.1	Q9M156
Cluster-10691.127683	471	UGT71	3.312E-164	68.6	Q6VAB2
Cluster-10691.201491	332	UGT71	1.1949E-86	62.0	D3THI6

Cluster-10691.79338	355	UGT708	3.6533E-80	60.3	A0A0A1HA03
Cluster-12940.0	414	UGT708	1.7E-111	62.9	A0A0A1HA03
Cluster-10691.102394	314	UGT80	0	86.8	Q9XIG1
Cluster-10691.50439	559	UGT80	0	82.7	Q9XIG1
Cluster-10691.51186	451	UGT74	2.526E-147	67.4	K7NBW3
Cluster-18386.0	369	UGT74	1.9366E-76	59.7	K7NBW3
Cluster-32153.0	453	UGT74	1.01E-152	66.7	K7NBW3
Cluster-32154.0	452	UGT74	5.641E-148	66.8	K7NBW3
Cluster-9689.1	408	UGT74	1.2371E-98	62.1	K7NBW3
Cluster-9689.2	408	UGT74	1.0039E-94	60.5	K7NBW3
Cluster-9689.4	405	UGT74	1.4901E-96	60.6	K7NBW3
Cluster-13980.0	439	UGT78	1.889E-156	66.0	Q9LFJ8
Cluster-10691.108718	472	UGT85	0	78.4	F8WKW1
Cluster-10691.119776	310	UGT85	1.349E-136	75.7	F8WKW1
Cluster-10691.73743	468	UGT85	0	82.8	F8WKW1
Cluster-10691.123734	310	UGT709	3.381E-125	73.0	U3U992
Cluster-10691.45154	319	UGT709	6.526E-149	81.6	U3U992
Cluster-10691.55310	483	UGT709	9.422E-176	70.0	U3U992
Cluster-10691.82758	420	UGT709	2.415E-162	70.8	U3U992
Cluster-10691.98625	495	UGT709	0	77.0	U5NH37
Cluster-10691.162684	309	UGT85	2.327E-127	75.3	B2XBQ5
Cluster-32849.0	309	UGT85	2.327E-127	75.3	B2XBQ5

Supplement Table S6. qRT-PCR primer name and sequences.

	Gene ID	Sequence（5′-3′）
H. ammodendron	Cluster-20042.94036	GCCACTATCGGGACAACCTC
		AGGAACCATTTGTGGGCATTC
	Cluster-20042.67782	GGTGGTATCTCTACTTCTTTGAGAG
		TCCCAATTCCAGTAGGGCAC
	Cluster-5748.0	CCACAAGCAAGACAACTTCCA
		ATCCCCATCTGGCTTTTTCGT
	Cluster-20042.44994	GTTTGGGCGATAGGTCGTGA
		CGATGCAAACCCGTCTACCT
	Cluster-31032.0	GCGCAGTTTGAAGCAGGTTT
		ACGAGTTCCTACTGCTGTTGA
	Internal reference	CAACTGGCTTCAAATGTGGTATCA
		ATCTTCACGGGCTTCAGAAAACTC
C. deserticola	Cluster-20042.72273	CCAGAGCACTTCGTATGGGA
		GGTAGCAAGAAGCATGACGG
	Cluster-20042.72232	GGATCGAGTTATCGGGCAGG
		GGGGCAACATAAGAGGGGTT
	Cluster-20042.72233	ATGGAACCCGACTTTCTCCC
		AGAAATCCACCTCTCGGGCT
	Cluster-20042.72797	TTGGCTTTCGCCACTAACCT
		GCGCCAACCCCTATCCAATA
	Cluster-20042.70944	GCCGACTACAACGAGGGATT
		CGGGTAGAAAAGCCCGAAGT
	Internal reference	TGTTGCTGGAGTGTTTGAGC

AACCAAGGACTTGCGAACAG
